# Immunomodulatory properties of *Leishmania tarentolae* extracellular vesicles containing the Spike protein of SARS-CoV-2

**DOI:** 10.3389/fpara.2024.1306478

**Published:** 2024-04-08

**Authors:** Ana Catalina Medina, Hamlet Acevedo Ospina, Albert Descoteaux

**Affiliations:** ^1^INRS- Centre Armand-Frappier Santé Biotechnologie, Université du Québec, Laval, QC, Canada; ^2^Infectiopôle INRS, Laval, QC, Canada

**Keywords:** *Leishmania*, Spike protein, SARS-CoV2, extracellular vesicles, dendritic cells, macrophages

## Abstract

Extracellular vesicles released by the protozoan parasite *Leishmania* display immunomodulatory properties towards mammalian immune cells. In this study, we have evaluated the potential of extracellular vesicles derived from the non-pathogenic protozoan *Leishmania tarentolae* towards the development of a vaccine adjuvant. As a proof of concept, we expressed in *L. tarentolae* a codon-optimized SARS-CoV-2 Spike protein fused to the *L. mexicana* secreted acid phosphatase signal peptide in the N-terminal and to a 6×-His stretch in the C-terminal. Extracellular vesicles released by the engineered *L. tarentolae* were isolated by ultracentrifugation and fast protein liquid chromatography and were characterized via nanoparticle tracking analysis and transmission electron microscopy. The recombinant S protein was present in extracellular vesicles released by *L. tarentolae*, as determined by Western blot analyses and immunoelectron microscopy. Next, we evaluated the immunomodulatory potential of extracellular vesicles containing the S protein towards bone-marrow-derived macrophages and bone-marrow-derived dendritic cells. Our data show that in bone-marrow-derived dendritic cells, extracellular vesicles containing the S protein induced an increased expression of proinflammatory genes compared to plain extracellular vesicles whereas the opposite was observed in bone-marrow-derived macrophages. These findings reveal the immunomodulatory potential of *L. tarentolae* extracellular vesicles and provide a proof of concept that they can be used as adjuvant in the context of dendritic cell stimulation.

## Introduction

Extracellular vesicles (EVs) are spherical lipid bilayer-enclosed particles released from all cell types, ranging in size from 30 to 5,000 nm in diameter (Willms et al., 2018; Verweij et al., 2021). They contain biologically active cargos, including macromolecules such as proteins, nucleic acids, and lipids, as well as metabolites and small molecules (Battistelli and Falcieri, 2020; Huda and Nurunnabi, 2022). These vesicles display highly dynamic and heterogeneous contents and membrane composition, which depend on the cellular source, state, and environmental conditions (Yanez-Mo et al., 2015). Such features raised the possibility that EVs may possess immunomodulatory properties and therefore could act as adjuvants for vaccination (Jesus et al., 2018; Huda and Nurunnabi, 2022). Indeed, various studies have exploited those characteristics to use EVs as vaccinal platforms. For example, EVs released from immune cells carry major histocompatibility complex molecule (MHC) I and MHC II, and co-stimulatory and adhesion molecules, which can directly stimulate CD8+ and CD4+ T cells (Devhare and Ray, 2018; Jesus et al., 2018). Other strategies focused on EVs released by pathogens or infected cells. Thus, according to the intrinsic nature of EVs, they may contain virulence factors that act as pathogen-associated molecular patterns (PAMPs), thereby contributing to the activation of immune cells. Given these characteristics, EVs have been used as vaccination agents that copy the pathogen they are derived from (Alaniz et al., 2007; Del Cacho et al., 2016; Montaner-Tarbes et al., 2018; Pal et al., 2020). For instance, the immunogenicity of EVs from bacteria such as *Neisseria meningitis* serogroup B has already been harnessed in two licensed vaccines (Bai et al., 2011; Acevedo et al., 2014). EVs have also been used as vehicles to express immunomodulatory pathogen antigens. Hence, 293T cell-derived EVs expressing the Spike (S) protein from the severe acute respiratory syndrome (SARS) virus triggered a humoral response, including neutralizing antibodies (Kuate et al., 2007). In another study, it was reported that EVs expressing the S protein from SARS-CoV-2 evoked a stronger humoral response, which, however, did not give rise to neutralizing capability, or an effective T-cell response compared to an adenoviral vector DNA vaccine encoding for the S protein. Nonetheless, when they used EVs expressing the S protein as a booster after priming with DNA S-EVs, they obtained both types of protective responses (Polak et al., 2020), which was also observed by a different group (Kuate et al., 2007). It is important to note that no adjuvants were added in these studies since EVs used in the assay were obtained from human cells and these vesicles contain human proteins that might be targeted by the murine immune response.

*Leishmania* actively releases EVs that modulate mammalian immune cells both *in vitro* and *in vivo* and contribute to the exacerbation of lesions associated with cutaneous leishmaniasis (Silverman et al., 2010; Silverman and Reiner, 2012; Atayde et al., 2015; Dong et al., 2019; Dong et al., 2021). Proteomic characterization of *Leishmania*-derived EVs revealed that they carry a wide range of biological molecules with potent immunostimulatory properties (Silverman et al., 2008; Hassani and Olivier, 2013; Forrest et al., 2020; da Silva Lira Filho et al., 2022). The ability of *Leishmania*-derived EVs to modulate immune cell responses is therefore consistent with their potential use as a vaccine platform (Olivier and Fernandez-Prada, 2019; Dong et al., 2021). However, manipulation of pathogenic parasites and the use of EVs derived from these pathogens in vaccination studies raise a number of safety issues. In contrast to other *Leishmania* species, the lizard parasite *L. tarentolae* is not pathogenic to humans and mammals in general and has therefore been used in various biotechnological and biomedical applications (Klatt et al., 2019), including its use as a vaccine candidate against the human pathogen *L. donovani* in an experimental model of visceral leishmaniasis (Breton et al., 2005). In addition, *L. tarentolae* has been genetically modified to express antigens from pathogens including the human papillomavirus, the hepatitis C virus, and the SARS-CoV-2, and was tested as a live vaccine in mice (Mizbani et al., 2009; Mizbani et al., 2011; Salehi et al., 2012; Hosseinzadeh et al., 2013; Ansari et al., 2019; Varotto-Boccazzi et al., 2022).

In this study, we have evaluated the potential of EVs derived from *L. tarentolae* towards the development of a vaccine adjuvant. As a proof of concept, we have expressed the SARS-CoV-2 S protein (Corbett et al., 2020; Walls et al., 2020) in *L. tarentolae* and here we report on the evaluation of the immunomodulatory properties of EVs enriched in S protein towards bone-marrow-derived macrophages (BMMs) and bone-marrow-derived dendritic cells (BMDCs).

## Materials and methods

### Ethics statement

All animal handling was performed in accordance with protocols 1806–01 and 1806–02, which were approved by the *Comité Institutionel de Protection des Animaux* of the INRS-Centre Armand-Frappier Santé Biotechnologie. These protocols respect procedures on animal practice as instructed by the Canadian Council on Animal Care, described in the *Guide to the Care and Use of Experimental Animals*.

### Parasites

The *L. tarentolae* strain P10 was obtained from Jena Bioscience (Jena, Germany) and was cultured at 26°C in brain heart infusion (BHI, BD Bacto) medium supplemented with hemin (5 μg/mL) in the presence of penicillin (50 U/mL) and streptomycin (50 μg/mL). The *L. tarentolae* P10 strain is derived from the TARII/UC strain (Klatt et al., 2019).

### Expression vectors and molecular cloning

The full-length DNA sequence encoding the SARS-CoV-2 Spike glycoprotein was derived from the genomic sequence of the isolated “severe acute respiratory syndrome coronavirus 2 Wuhan-Hi-1” released in January 2020 (accession number MN908947). This sequence was codon-optimized for expression in *L. tarentolae* using the Sequence Manipulation Suite (www.bioinformatics.org/sms2/) Reverse Translate Tool and the *L. tarentolae* codon usage table. To retain the S protein in the antigenically optimal prefusion conformation (Pallesen et al., 2017; Wrapp et al., 2020), modifications at the S1/S2 cleavage site to confer resistance to furin were achieved by mutating the furin cleavage site 682-RRAR-685 to 682-GSAS-685. In addition, two proline substitutions at amino acid position K986–V987 were performed to maintain the prefusion state and increase immunogenicity (Pallesen et al., 2017). The DNA sequence was synthetically produced by BioBasic (Markham, Canada) and cloned into the plasmid pUC57 using *Sal*I and *Kpn*I. The 3,606-bp *Sal*I–*Kpn*I fragment was excised and cloned into the pLEXSY-hyg2.1 (Jena Bioscience) *Sal*I and *Kpn*I sites to generate pLEXSY-hyg2.1-rSpike. The resulting recombinant Spike (rS) protein contains an N-terminal signal peptide for secretory expression and a C-terminal His6 for protein identification and purification purposes.

### Transfection of the *Leishmania tarentolae* P10 strain

A 9.4-kb *Swa*I fragment from pLEXSY-hyg2.1-rSpike containing the expression cassette with the rS cDNA was gel-purified and transfected into *L. tarentolae* P10 by electroporation. This expression cassette vector integrates into the chromosomal 18S rRNA (*ssu*) locus of *L. tarentolae*, allowing for the constitutive expression of rS. Briefly, parasites at a density of 6 × 10^7^ cells/mL were resuspended at a concentration of 10^8^ cells/mL and kept on ice for 10 min. Five micrograms of the rS expression *Swa*I cassette was dissolved in 50 µL of dH_2_O and added to 350 µL of pre-chilled parasites and transferred to 2-mm electroporation cuvettes. Electroporation was performed with a Gene Pulser II (Bio-Rad Laboratories) as previously described (Descoteaux A et al., 1994). Electroporated promastigotes were placed on ice for 10 min and were transferred to a 10-mL BHI medium supplemented with hemin (5 μg/mL), penicillin (50 U/mL), and streptomycin (50 μg/mL). Cells were incubated overnight at 26°C as a static suspension culture before polyclonal selection, which was achieved in the presence of hygromycin (100 μg/mL) at 26°C. To assess the potential impact of rS expression on the growth of *L. tarentolae*, WT and rS-expressing *L. tarentolae* promastigotes were seeded in supplemented BHI medium at an initial density of 1 × 10^6^ parasites/mL and were enumerated every 24 h for 4 days.

### Analysis of rSpike expression

To analyze intracellular rS expression in *L. tarentolae*, parasites were seeded at 1 × 10^6^ parasites/mL and were grown at 26°C in BHI medium supplemented with 5 μg/mL hemin, 50 U/mL penicillin, and 50 μg/mL streptomycin (and 100 μg/mL hygromycin for rS-expressing *L. tarentolae*) until they reached a density of 9 × 10^6^ parasites/mL. We used 0.5-mL aliquots from parasite cultures. Cells were centrifuged for 7 min at 2,600*g* and pellets were resuspended in 0.2 mL of lysis buffer [1% NP-40, 500 mM Tris–HCl (pH 7.5), 150 mM NaCl, 1 mM EDTA (pH 8), 1.5 mM EGTA, 1 mM Na_3_VO_4_, 50 mM NaF, 10 mM Na_4_P_2_O_7_, 10 mM 1,10-phenanthroline, and protease inhibitors] and passed through a needle to help disrupt cells, membranes, and DNA. After incubation at −80°C for at least 30 min, samples were quantified and kept at −20°C for posterior use. For the analysis of rS secretion, sterile-filtered culture supernatants from cultures at 9 × 10^6^ parasites/mL were concentrated 100× by trichloroacetic acid (TCA) precipitation as follows. Cells were pelleted from 10 mL of culture supernatant by centrifugation for 10 min at 2600*g* and 8 mL of sterile-filtered supernatant was added to 2 mL of 50% ice-cold TCA to yield a final concentration of 10%. Sterile filtration of the supernatant prior to TCA precipitation was performed to avoid carryover of cells, and the supernatant was left on ice for 30 min before centrifugation for 15 min at 15,000*g* and 4°C. After removing the supernatant, the pellet was resuspended in 1 mL of 80% acetone and transferred to an Eppendorf tube (to remove the residual TCA). After an additional centrifugation for 15 min at 15,000*g* and 4°C, the supernatant was aspirated, and the pellet was resuspended in a final volume of 80 µL of lysis buffer for posterior use. Levels of rS were assessed by Western blot analysis as previously described (Guay-Vincent et al., 2022). Rabbit anti-SARS-CoV-2 (2019-nCoV) Spike receptor-binding domain (RBD) polyclonal antibody was from Sinobiological, mouse monoclonal anti-GP63 antibody #235 was kindly provided by Dr. W. Robert McMaster (University of British Columbia), and the mouse anti-penta-His polyclonal antibody was from Qiagen. Secondary horseradish peroxidase (HRP)-conjugated IgG anti-rabbit and anti-mouse antibodies were from Sigma. Hybond membranes were incubated in ECL (GE Healthcare) and immunodetection was achieved via chemiluminescence.

### Isolation of extracellular vesicles

*L. tarentolae* expressing rS or not were seeded at a density of 1 × 10^6^ promastigotes/mL in 10 mL of BHI (BD Bacto) medium supplemented with hemin (5 μg/mL), penicillin (50 U/mL), streptomycin (50 μg/mL) (Life Technologies), and hygromycin (100 μg/mL) and were incubated at 26°C for 3–4 days until they reached a density of 9 × 10^6^ parasites/mL. Cell culture medium was harvested, followed by the addition of protease inhibitor and 1,10-phenanthroline (Sigma) and filtration through a 0.22-μm membrane filter before following the procedure described by Vucetic et al. (2020). Briefly, the supernatant was ultracentrifuged at 100,000*g* and 4°C for 70 min, and the pellet was washed two times with exosome buffer (137 mM NaCl and 20 mM HEPES, pH 7.5). The EV pellet was resuspended in approximately 150–200 μL of exosome buffer, and the samples were quantified by Nanodrop One and aliquoted. Samples were stored at −80°C.

#### Enrichment of rSpike-enriched extracellular vesicles

*L. tarentolae* expressing rS were seeded at a density of 1 × 10^6^ promastigotes/mL in 50 mL of BHI (BD Bacto) medium supplemented with 5 μg/mL hemin, 50 U/mL penicillin, 50 µg/mL streptomycin, and 100 μg/mL hygromycin and were incubated at 26°C for 3–4 days until they reached a density of 1 × 10^8^ parasites/mL. EVs enriched in rS (EV-rS+) were obtained by direct purification of supernatants (50 mL) through 5-mL high-performance immobilized metal affinity chromatography (IMAC) HisTrap HP His tag protein purification columns (Cytiva) for His tag recombinant protein purification in a fast protein liquid chromatography system (ÄKTA FPLC Systems, GE Healthcare). The binding capacity of these columns is 40 mg of His tag protein per milliliter of resin. The column was equilibrated with 50 mM NaH_2_PO_4_ and 300 mM NaCl (pH 8), and then fractions were obtained using elution buffer (50 mM NaH_2_PO_4_, 300 mM NaCl, and 500 mM imidazole, pH 8) using a linear gradient of up to 100% imidazole with a constant flow of 1 mL/min. The presence of rS in each fraction was assessed by Western blot analyses and five fractions with the highest levels of rS+ (EV-rS+) were pooled prior to quantification by Nanodrop One and characterization by nanoparticle tracking analysis (NTA; described below). We routinely obtained a yield of 3.0 µg of purified EV-rS+ per milliliter of *L. tarentolae* expressing rS conditioned medium.

### rSpike purification

Purification of rS by IMAC was achieved from cultures at 9 × 10^6^ parasites/mL concentrated 100× by TCA precipitation with a modification in the equilibrium buffer with urea (6 M urea, 50 mM NaH_2_PO_4_, and 300 mM NaCl, pH 8). Fractions were obtained as described above using elution buffer (50 mM NaH_2_PO_4_, 300 mM NaCl, and 500 mM imidazole, pH 8). Samples were then dialyzed, and a Western blot was run to confirm the presence of rS.

### Transmission electron microscopy

Transmission electron microscopy (TEM) analysis was performed at the Service de Microscopie Electronique of INRS-Armand-Frappier Santé Biotechnologie on EVs derived from *L. tarentolae*. Grids were placed on a parafilm and a 10-μL drop of EV suspension was deposited for 20 min. Using a piece of bibulous paper, grids were dried, immersed in a drop of 5% uranyl acetate in 50% ethanol for 1 min, and dried again. Imaging was performed on a Hitachi H7100 transmission electron microscope with an AMT Camera (XR-100) at 75 kV.

### Immunogold staining

For immunogold labeling, EVs released by *L. tarentolae* expressing rS or not were fixed and embedded as follows: fixation in 0.1% glutaraldehyde + 4% paraformaldehyde in a cacodylate buffer at pH 7.2 overnight, and dehydration by successive passages through 25%, 50%, 75%, and 95% solutions of acetone in water for 30 min each, followed by immersion in two changes of pure acetone for at least 30 min each. Samples were immersed for 16–18 h in SPURR (TedPella):acetone (1:1), embedded by immersion in two successive baths of SPURR (TedPella) for at least 2 h each, cut into small pieces, placed in BEEM capsules (TedPella) that were filled with SPURR mixtures, and left to stand at room temperature for 18 h. Afterwards, the filled capsules were placed at 60°C for 24 h to polymerize the resin before the samples were cut using an ultramicrotome system (Leica UC7) and put onto Formvar-coated (EMS) and carbon-coated (Leica ACE600) nickel 200 mesh grids (EMS). After cutting the sample with the ultramicrotome, samples were placed on nickel grids treated with a saturated solution of sodium metaperiodate and blocked with 1% bovine serum albumin in phosphate-buffered saline. Grids were then incubated with rabbit polyclonal SARS-CoV-2 (2019-nCoV) Spike RBD antibody (1:2,000 dilution, Sinobiological), washed, and incubated with 20 nm of anti-rabbit gold particle-conjugated secondary antibody (Abcam). After washing, samples were contrasted with uranyl acetate and lead citrate and subsequently visualized using a Hitachi H7100 transmission electron microscope.

### Nanoparticle tracking analysis

The concentration and size distribution of EVs were measured using NTA on a Nanosight NS300 instrument (Malvern Instruments Ltd., Malvern, UK). Fractions were diluted 1,000- to 4,000-fold in water. Each sample was captured in triplicate for 30 s with the camera level set to 14 and a detect threshold of 5. The data were analyzed using the NTA software (version 3.4 build 3.4.003).

### Mammalian cell culture

Bone marrow was flushed from the femurs and tibias of 6- to 12-week-old 129/BL6 mice and treated with 0.17 M NH_4_Cl, pH 7.4, for 7 min to lyse red blood cells. To generate BMMs, marrow cells were differentiated for 7 days in complete DMEM containing L-glutamine (Life Technologies), 10% v/v heat-inactivated fetal bovine serum (FBS) (Life Technologies), 10 mM HEPES (Bioshop) at pH 7.4, and penicillin–streptomycin (Life Technologies) supplemented with 15% v/v L929 cell-conditioned medium (LCM) as a source of macrophage colony-stimulating factor-1 (CSF-1). BMMs were made quiescent prior to use by culturing them in the absence of CSF-1 for 24 h (Descoteaux and Matlashewski, 1989). BMDCs were differentiated for 7 days in complete RPMI (Life Technologies), supplemented with 10% v/v heat-inactivated FBS (Life Technologies), 10 mM HEPES (Bioshop) at pH 7.4, and penicillin–streptomycin (Life Technologies) and 10% X63 cell supernatants as a source of granulocyte-macrophage colony-stimulating factor (GM-CSF). On day 3, the medium was refreshed. On day 7, non-adherent cells were harvested in RPMI supplemented with FBS 10% and 5% X63 supernatants. BMMs and BMDCs were grown and differentiated in a humidified 37°C incubator with 5% CO_2_ (Arango Duque et al., 2021).

### *In vitro* stimulation of BMMs and BMDCs

BMMs or BMDCs were stimulated for 6 h with 1, 10, and 100 μg/mL of either EV-WT, EV-rS+, or purified S protein. Cells treated with 100 ng/mL of LPS were used as a positive control, and unstimulated cells were used as a negative control. After 6 h, cells were washed with Hanks’ balanced salt solution (HBSS) twice and RLT lysis buffer from the RNeasy kit (Qiagen) was added. Samples were kept at −80°C until the RNA extraction was performed.

### Gene expression analyses

Total RNA from BMMs and BMDCs was extracted with the RNeasy^®^ Mini Kit (Qiagen) following the manufacturer’s instructions. RNA (500 ng) was reverse transcribed to cDNA using the iScript cDNA Synthesis Kit (Bio-Rad Laboratories). cDNAs were analyzed using Agilent Technologies Stratagene Mx3000P or QuantStudio3 Applied biosystems qPCR for interleukin-6 (*IL-6*), interleukin-10 (*IL-10*), interleukin-12 (*IL-12*), tumor necrosis factor (*TNF*), interleukin-1α (*IL-1α*), and interleukin-1β (*IL-1β*) genes using ribosomal protein S29 as a housekeeping gene. The 2^−ΔΔCt^ method was applied to compare the relative expression between the target gene and the housekeeping gene.

### Statistical analyses

Statistical analyses were performed using GraphPad Prism 6. Statistical differences between the evaluated groups were determined with one-way ANOVA, then each group was compared using an unpaired *t*-test. Each experiment was performed in triplicate and a *p*-value of <0.05 was considered as statistically significant.

## Results

In the present study, we have evaluated the potential of EVs derived from *L. tarentolae* towards the development of a vaccine adjuvant. In the first part, we have expressed in *L. tarentolae* promastigotes an engineered SARS-CoV-2 Spike (rS) protein fused to the *L. mexicana* secreted acid phosphatase signal peptide in the N-terminal for secretion. We showed that the engineered rS protein is present in both EVs and EV-depleted exoproteome. In the second part, we assessed the potential of EVs expressing rS to stimulate the expression of genes encoding proinflammatory cytokines in BMMs and BMDCs.

### The SARS-CoV-2 S protein expressed by *L. tarentolae* promastigotes is associated with extracellular vesicles

**Figure 1:**
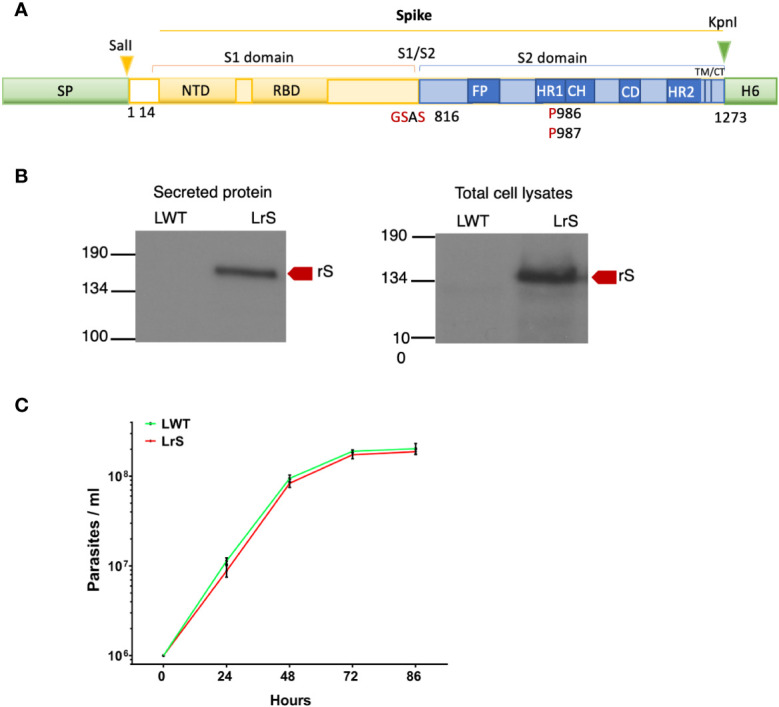
Expression of the SARS-CoV-2 Spike protein in *L. tarentolae.* **(A)** Schematic representation of wild-type Spike. Shown in red are substitutions of K986 and V987 by two prolines and mutation in the S1/S2 furin cleavage site RRAR for GSAS. **(B)** The presence of rS in supernatants and total cell lysates from LWT and LrS was assessed by Western blot analysis using an anti-penta-His antibody. Fifteen micrograms of TCA-precipitated supernatants and 15 μg of total cell lysates were loaded for each sample. **(C)** Expression of rS has no significant effect on *L. tarentolae* growth. LWT and LrS were seeded at 1 × 10^6^ parasites/mL and growth was monitored every 24 h for 4 days. The graph represents the means of two independent experiments performed in triplicate. SP, signal peptide; NTD, N-terminal domain; RBD, receptor-binding domain; FP, fusion peptide; TM, transmembrane domain; CT, cytoplasmic domain; H6, hexahistidine; rS, recombinant Spike protein, LWT, *L. tarentolae* WT, LrS, *L. tarentolae* expressing rS.

**Figure 2:**
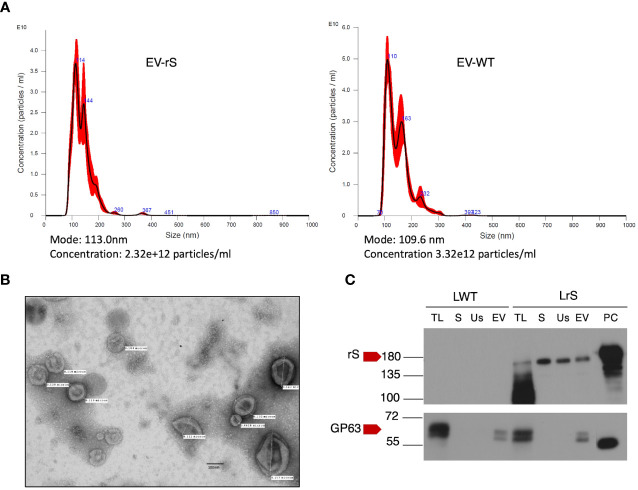
Characterization of EVs released by *L. tarentolae.* **(A)** Concentration and size distribution of EVs recovered from the supernatants of LWT (EV-WT) and LrS (EV-rS) were measured using nanoparticle tracking analysis (NTA) on a Nanosight NS300. **(B)** EV-rS were imaged by transmission electron microscopy at 75 kV and 40,000× magnification. **(C)** The presence of S protein and GP63 in EV-WT and EV-rS was evaluated by Western blot analysis using an anti-S RBD antibody and an anti-GP63 antibody, respectively. Fifteen micrograms of protein was loaded for each sample. LWT, *L. tarentolae* WT; LrS, *L. tarentolae* expressing rS; TL, total cell lysate; S, supernatant; Us, ultracentrifugation supernatant; EV, extracellular vesicle; PC, positive control for rS; rS, recombinant Spike protein.

**Figure 3:**
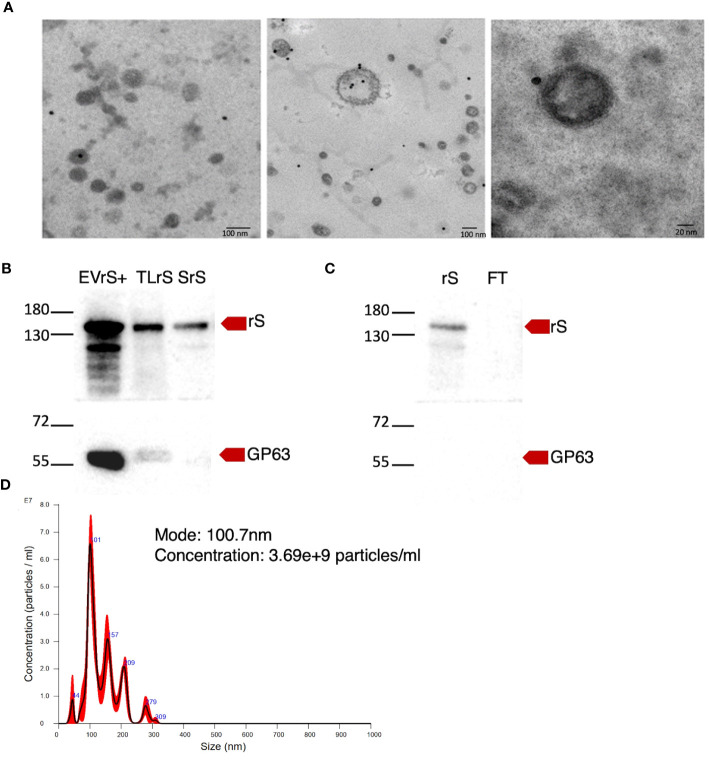
Enrichment of EVs expressing rS. **(A)** The presence of rS in EVs released by *L. tarentolae*-expressing rS (LrS) was assessed by immunoelectron microscopy using an anti-RBD antibody and a secondary antibody coupled to 20-nm gold particles. TEM images were recorded at a voltage of 75 kV and at 40,000× magnification for the left and middle panels and at 100,000× magnification for the right panel. **(B)** EVs containing rS released by LrS were enriched by FPLC and characterized by Western blot. Fifteen micrograms of protein was loaded for each sample. **(C)** rS present in the supernatant of LrS was purified under denaturing conditions by FPLC. Fifteen micrograms of protein was loaded for each sample. Rabbit polyclonal SARS-CoV-2 (2019-nCoV) Spike RBD antibody (1:2,000) was used to identify rS and mouse monoclonal GP63 antibody (1:10) was used as an EV marker. **(D)** EV-rS size after enrichment was measured by NTA. EVrS+, EVs containing recombinant Spike; TLrS, total lysate from *L. tarentolae* expressing rS; SrS, secreted protein from *L. tarentolae* expressing rS; rS, recombinant Spike; FT, flow-through.

We stably expressed in *L. tarentolae* a codon-optimized SARS-CoV-2 S protein fused to the *L. mexicana* secreted acid phosphatase signal peptide in the N-terminal and to a 6×-His stretch in the C-terminal (rS) (**Figure 1A**). Using an anti-His antibody, we detected by Western blot a protein of approximately 180 kDa in the supernatant and cell lysates of rS-expressing *L. tarentolae* (LrS) promastigotes (**Figure 1B**), confirming that rS is expressed and secreted by *L. tarentolae*. As shown in **Figure 1C**, expression of rS had no significant impact on the growth of *L. tarentolae* promastigotes. In *Leishmania*, a large number of secreted proteins are associated with EVs (Silverman et al., 2008; Hassani and Olivier, 2013; Forrest et al., 2020; da Silva Lira Filho et al., 2022). Therefore, we next assessed whether rS is present in EVs released by *L. tarentolae* promastigotes. To this end, we isolated EVs by ultracentrifugation from the supernatants of wild type (LWT) and LrS *L. tarentolae* promastigotes cultures. EVs released by both LrS and LWT were comparable in size and concentration, as determined by NTA (**Figure 2A**). Additionally, EVs released by LrS and LWT were examined by TEM to confirm their size and were found to display the typical round morphology of EVs (**Figure 2B**). We next confirmed by Western blot analysis that rS was present in EVs released by LrS but not by LWT promastigotes, whereas we detected the *Leishmania* exosomal marker GP63 in both populations of EVs, in agreement with the nature of these vesicles (Hassani and Olivier, 2013; Forrest et al., 2020; Shokouhy et al., 2022) (**Figure 2C**). Of note, rS was also present in post-ultracentrifugation supernatants, indicating that this protein is present in both EVs and in the EV-depleted exoproteome. Having established that a significant proportion of rS secreted by *L. tarentolae* promastigotes is associated with EVs, we further characterized these EVs by immunogold electron microscopy. As shown in **Figure 3A**, we observed a heterogenous distribution of rS in the EVs isolated by ultracentrifugation. To enrich the proportion of EVs containing rS (EV-rS), we took advantage of the presence of the His tag at the C-terminal to purify these EVs using IMAC. As shown in **Figure 3B**, we detected GP63 (band at approximately 63 kDa) and rS (band at approximately 180 kDa) in these EVs. These EV preparations isolated by IMAC were enriched in rS, which co-purified with EVs containing rS and therefore represent EV-enriched fractions. We also used IMAC to purify rS from EV-depleted supernatants of LrS (**Figure 3C**). The anti-GP63 antibody was used as a control to verify the purity of the rS protein. NTA of the affinity-purified EV-rS (EV-rS+) showed a more heterogeneous population compared to the EVs obtained by ultracentrifugation, with a mean of 139.7 nm and a mode of 100.7 nm in size, keeping 90% of EVs below 210.9 nm (**Figure 3D**).

### Proinflammatory potential of *L. tarentolae* EVs

**Figure 4:**
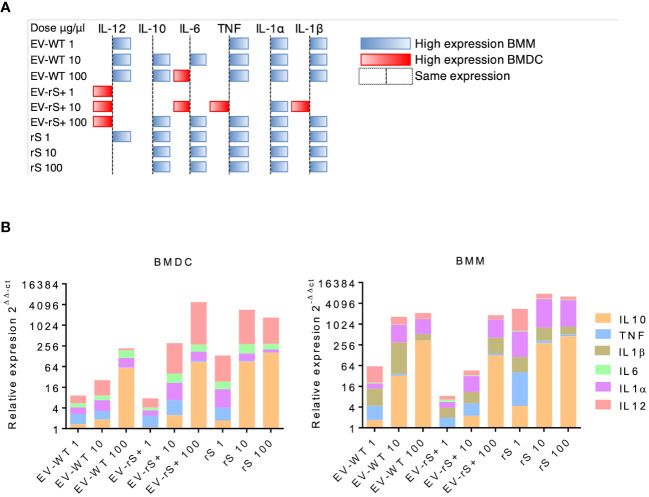
Differential cytokine gene expression in BMMs and BMDCs exposed to EV. **(A)** BMMs and BMDCs were stimulated with different doses of EVs expressing rS (EV-rS) or not (EV-WT) and recombinant Spike alone (rS) for 6 h. The cytokine gene expression was determined using RT-qPCR. The heat map was built using the highest 2^−ΔΔCt^ value for each cytokine. When the 2^−ΔΔCt^ value was higher on BMMs, it was represented as blue; when the 2^−ΔΔCt^ value was higher on BMDCs, it was represented as red. When they showed a similar expression, no colors were used. **(B)** Dose-dependent effect in BMDCs and BMMs: The inflammatory potential of EVs expressing recombinant S (EV-rS) was evaluated by quantifying by qRT-PCR the expression of cytokine genes such as *IL-6*, *IL-10*, *IL-12*, *TNF*, *IL-1α*, and *IL-1β in vitro* in BMDCs and BMMs after 6 h of stimulus with EV-rS, EV that does not express S (EV-WT), and recombinant S (rS).

**Figure 5:**
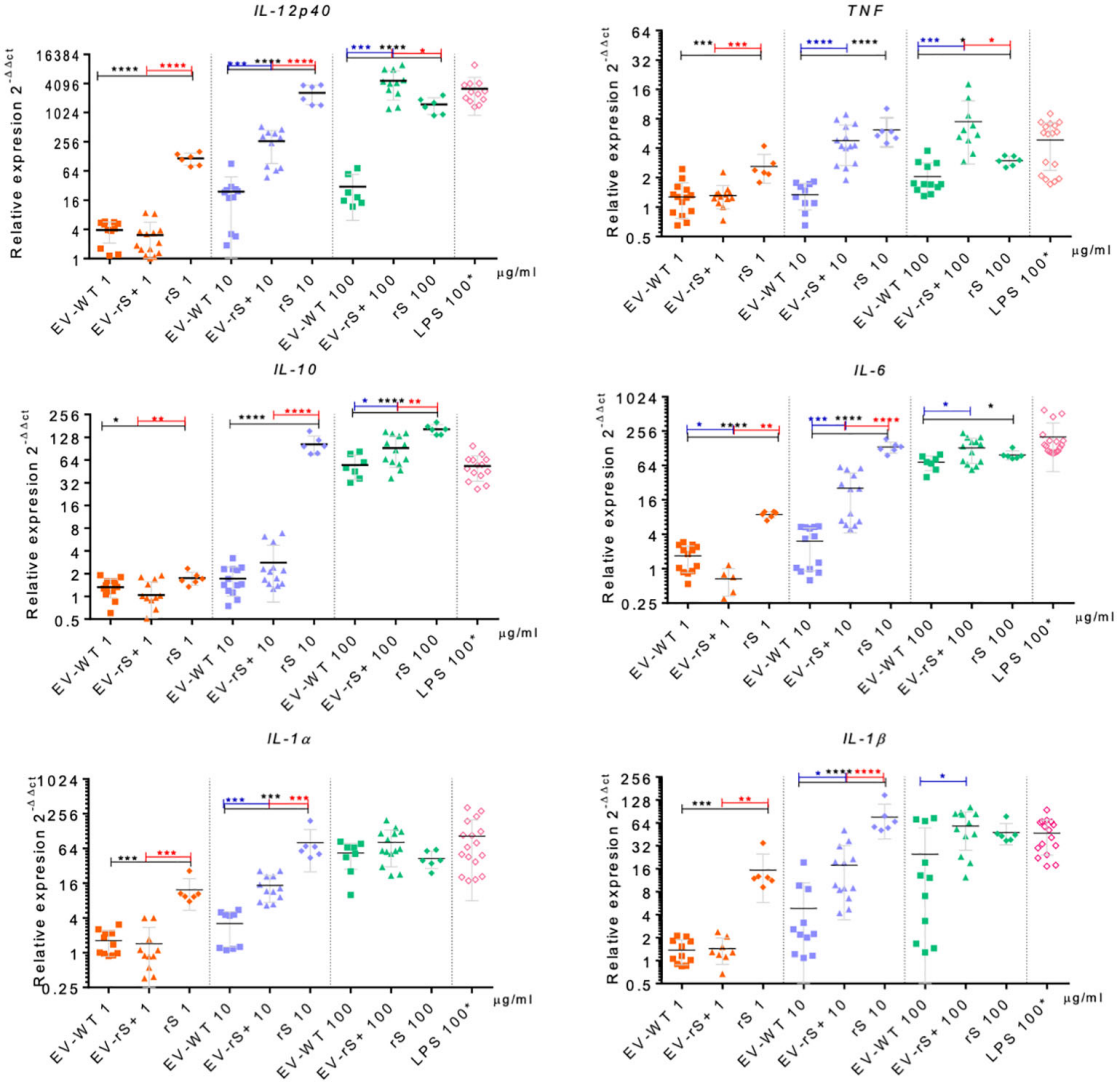
Immunostimulatory effect of EVs and Spike towards BMDCs. BMDCs were incubated in the absence or presence of 1, 10, or 100 μg/mL EV-WT, EV-rS+, and rS for 6 h. Positive controls consisted of BMDCs incubated for 6 h with 100 ng/mL LPS. Total RNA was extracted and expression of *IL-12p40*, *TNF*, *IL-10*, *IL-6*, *IL-1α*, and *IL-1β* was assessed by qRT-PCR. Statistical differences between the groups were evaluated using one-way ANOVA, then each stimulus was compared using a two-tailed, unpaired Student’s *t*-test for each dose. Each experiment was performed in triplicate and a *p*-value of <0.05 was considered statistically significant. *, *p* from 0.01 to 0.05; **, *p* from 0.001 to 0.01; ***, *p* from 0.0001 to 0.001; ****, *p <*0.0001.

**Figure 6:**
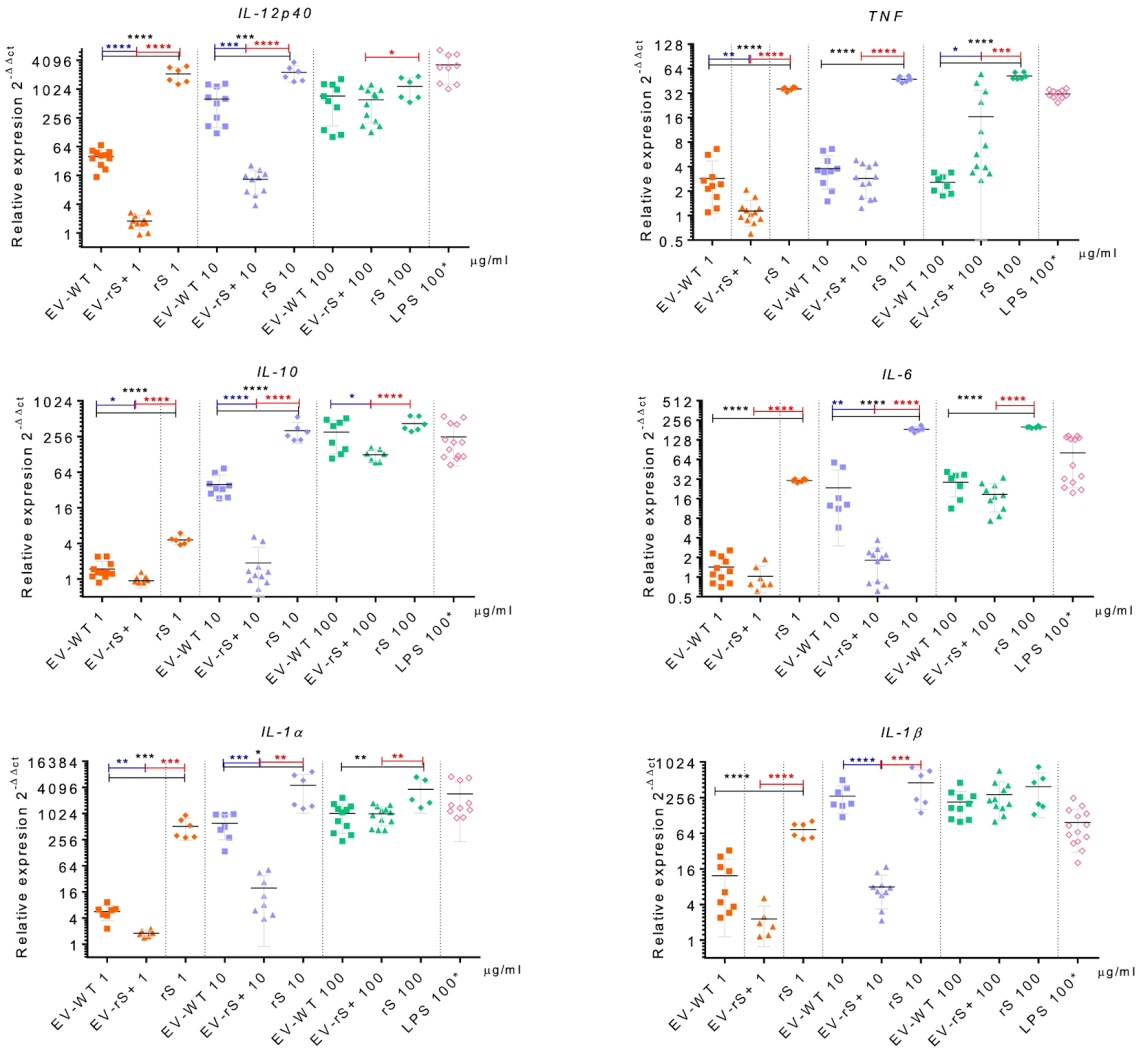
Immunostimulatory effect of EVs and Spike towards BMMs. BMMs were incubated in the absence or presence of 1, 10, or 100 μg/mL EV-WT, EV-rS+, and rS for 6 h. Positive controls consisted of BMMs incubated for 6 h with 100 ng/mL LPS. Total RNA was extracted and expression of *IL-12p40*, *TNF*, *IL-10*, *IL-6*, *IL-1α*, and *IL-1β* was assessed by qRT-PCR. Statistical differences between the groups were evaluated using one-way ANOVA, then each stimulus was compared using a two-tailed, unpaired Student’s *t*-test for each dose. Each experiment was performed in triplicate and a *p*-value of <0.05 was considered statistically significant. *, *p* from 0.01 to 0.05; **, *p* from 0.001 to 0.01; ***, *p* from 0.0001 to 0.001; ****, *p <*0.0001.

Vaccines mimic pathogen infections, and our aim was to explore the potential of the *L. tarentolae* EV-based platform for vaccine development with the dual function of expression of antigen candidates and adjuvant stimulus. We first assessed the *in vitro* inflammatory potential of the EV-rS+ in terms of stimulating cytokine gene expression in macrophages and dendritic cells, given the role of these immune cells in the activation of the adaptive immune system and in enhancing antigen presentation (Awate et al., 2013; Correa et al., 2022). To this end, we stimulated BMMs and BMDCs with EVs purified from both wild-type *L. tarentolae* (EV-WT) and rS-expressing *L. tarentolae* (EV-rS+). We used rS purified from *L. tarentolae* supernatant as a stimulus to evaluate the effect of the protein without adjuvant and LPS as a positive control to stimulate a proinflammatory cytokine response. We used three different concentrations for each stimulus (1 μg/mL, 10 μg/mL, and 100 μg/mL), and we quantified the relative expression of genes encoding interleukin-12p40 (*IL-12B*), *IL-10*, *IL-6*, *IL-1α*, *IL-1 β*, and *TNF* after 6 h by RT-qPCR. **Figure 4** shows that EV-WT and rS induced a higher expression of cytokine genes in BMMs (blue boxes) whereas EV-rS+ induced higher cytokine gene expression in BMDCs (red boxes), in a dose-dependent manner. In BMDCs, we observed that purified rS (from 1 to 100 μg/mL) by itself potently stimulates the expression of all cytokine-encoding genes evaluated (**Figure 5**). Stimulation of BMDCs with EVs revealed dose-dependent immunostimulatory properties that were significantly superior for EV-rS+ compared to EV-WT, notably at a concentration of 10 μg/mL. At that concentration, EV-rS+ induced higher expression levels for all cytokine genes evaluated than EV-WT, with the exception of *IL-10*. At a concentration of 1 μg/mL, both EV-WT and EV-rS+ induced cytokine gene expression to a similar extent. In BMMs, we also found that rS stimulates the highest levels of cytokine gene expression at all concentrations evaluated (**Figure 6**). Interestingly, we found that stimulation with EV-rS+ reduced the expression of the six cytokine genes evaluated compared to EV-WT, especially at 1 and 10 μg/mL. When we evaluated the differences between the three stimuli at each concentration, we observed the highest differences at 1 μg/mL and 10 μg/mL for all the cytokines evaluated. Nonetheless, at 100 μg/mL, EVs induce similar relative expression of *IL-12A*, *IL-6*, *IL-1α*, and *IL-1β* cytokine genes. Notably, when we evaluated *IL-1β* expression, all stimuli induced similar levels at 100 μg/mL (**Figure 6**).

## Discussion

In the present study, we have investigated the immunomodulatory properties of EVs released by *L. tarentolae* promastigotes engineered to express a secreted form of the SARS-CoV-2 S (rS) protein. We observed that both EV-WT and EV-rS+ stimulated the expression of proinflammatory genes in BMDCs and BMMs. In BMDCs, EV-rS+ induced an increased expression of proinflammatory genes compared to EV-WT whereas the opposite was observed in BMMs. These findings point to the immunomodulatory potential of EVs produced by *L. tarentolae* and provide a proof of concept that they can be used as adjuvant in the context of BMDC stimulation.

Our results revealed that rS is secreted by *L. tarentolae*, consistent with the presence of the signal peptide from *L. mexicana* secreted acid phosphatase. Further analysis of the supernatant indicated that a significant portion of rS is associated with EVs. However, immunogold staining performed on unenriched EV-rS preparations revealed that not all EVs contained rS. For this reason, we performed an additional purification step using IMAC to obtain EVs enriched in rS (EV-rS+). Additional work may be necessary to improve the levels of rS in EVs released by *L. tarentolae*. One possible way is to design a fusion of the S protein or one of its subdomains with a component of *Leishmania*-derived EVs (Silverman et al., 2010; Hassani et al., 2014; Forrest et al., 2020). A similar approach was recently used by Kuate and collaborators, where the SARS full-length S was fused to the vesicular stomatitis virus-glycoprotein (VSV-G) to be expressed in a membrane-bound form, thus improving the antigen expression on EVs (Kuate et al., 2007). Other methodologies have been utilized to express antigen on the surface of EVs, such as DSPE-PEG-NHS linker use, which was conjugated to the RBD of the SARS-CoV-2 S protein (Wang et al., 2022).

Analysis of the secretome of *L. donovani* promastigotes revealed that less than 2% of the proteins contained an N-terminal classical secretion signal peptide, indicating that nonclassical secretion pathways are the dominant means by which *Leishmania* proteins are secreted (Silverman et al., 2008). Further studies based on comparative quantitative proteomics (Silverman et al., 2010) indicated that over 50% of the *Leishmania* secretome was present in EVs, which may represent a general mechanism for protein secretion in *Leishmania*. In the present study, we engineered rS to be secreted through a classical secretion pathway with the addition in the N-terminal of the signal peptide of the *L. mexicana* secreted acid phosphatase. Our data showed that despite the signal peptide, rS was also present in EVs. This observation is consistent with the notion that sorting of released proteins in *Leishmania* EVs is not influenced by the presence of canonical signal peptides. Nonetheless, the possibility that rS associates with EVs like a corona protein cannot be fully excluded.

EVs released by microorganisms contain a plethora of components with the potential to modulate innate and adaptive immune responses (Choi et al., 2015; Jesus et al., 2018; Perez-Cabezas et al., 2018). In this regard, our data showed that EVs released by *L. tarentolae* display proinflammatory properties. Hence, *L. tarentolae*-derived EV-WT and EV-rS+ both stimulated the expression of genes encoding proinflammatory cytokines in BMDCs, including *IL-12B, IL-1a*, *IL-1b*, *IL-6*, and *TNF*. Interestingly, we observed that in BMMs, EV-WT induced significantly higher mRNA levels for those cytokines than EV-rS+. We currently have no explanation for this difference between BMDCs and BMMs. Clearly, additional investigations will be necessary to understand the underlying mechanisms. In addition, future studies will be required to address the potential impact of proteins present in the culture medium BHI (Le et al., 2023), which might bind to *L. tarentolae*-derived EVs, on the expression of genes encoding proinflammatory cytokines in BMDCs and in BMMs. Consistent with our findings, a recent study on the characterization of EVs released by *L. tarentolae* revealed that stimulation of PMA-differentiated THP-1 macrophages with those EVs led to the increased release of IFN-γ, TNF, and IL-1β following infection with *L. major*, compared to THP-1 cells pretreated with EVs derived from *L. major* (Shokouhy et al., 2022). Interestingly, pretreatment of THP-1 cells with *L. tarentolae*-derived EVs strongly impaired the capacity of *L. major* to replicate in these cells. The use of *L. tarentolae* as a live vaccine revealed their ability to target dendritic cells and secondary lymphoid organs, and to induce a Th1 response (Breton et al., 2005; Shokouhy et al., 2022; Varotto-Boccazzi et al., 2022). This is in contrast to their pathogenic *Leishmania* species counterparts that tend to skew the immune response towards Th2, which allows multiplication and parasite survival. Interestingly, a recent study revealed that *L. tarentolae* did not induce polarization towards a distinctive Th1 or Th2 profile, which agrees with our preliminary results. Nonetheless, they noted a moderate immune polarization on the Th1 side by the expression of IL-2, IL-12, IFN-γ, and transcriptional factors (STAT1 and STAT4) upon infection of dendritic cells with *L. tarentolae* expressing the SARS-CoV-2 full-length S (Varotto-Boccazzi et al., 2022). In our model, there was not such an effect since EV-WT and EV-rS stimulated both pro- and anti-inflammatory cytokines’ gene expression.

We showed that rS is present in EVs derived from *L. tarentolae* engineered to express the SARS-CoV-2 Spike protein. We have also evaluated the immunomodulatory potential of these EVs towards macrophages and dendritic cells. Future studies need to evaluate the adjuvant potential of EV-rS by immunizing mice and to investigate their ability to elicit neutralizing antibodies towards SARS-CoV-2. In this regard, the use of EVs or exosomes as a vehicle to deliver SARS-CoV-2 Spike for vaccination purposes has been proposed and tested in other systems (Choi et al., 2022; Jiang et al., 2022; Cacciottolo et al., 2023a; Cacciottolo et al., 2023b; Kim and Thapa, 2023). In one of those studies, Cacciottolo and colleagues (Cacciottolo et al., 2023a) have expressed the Delta variant Spike linked to the N-terminal of the exosome-specific tetraspanin CD9 by a synthetic transmembrane domain and a secretion signal peptide. Immunization of mice with these Delta Spike-expressing EVs resulted in the robust generation of potent neutralizing antibodies against SARS-CoV-2 variants Delta and Omicron. This study constitutes a proof of concept for the use of Spike-expressing EVs as a novel vaccinal approach. Given the strong immunomodulatory properties of *Leishmania*-derived EVs (Atayde et al., 2016; Olivier and Fernandez-Prada, 2019), we can speculate that *L. tarentolae* EVs enriched in rS have a promising potential to elicit a potent immune response.

## Data availability statement

The raw data supporting the conclusions of this article will be made available by the authors, without undue reservation.

## Ethics statement

The animal study was approved by Comité Institutionel de Protection des Animaux of the INRS-Centre Armand-Frappier Santé Biotechnologie. The study was conducted in accordance with the local legislation and institutional requirements.

## Author contributions

AM: Conceptualization, Data curation, Formal Analysis, Investigation, Methodology, Writing – original draft, Writing – review & editing. HA: Formal Analysis, Writing – review & editing, Investigation. AD: Formal Analysis, Writing – review & editing, Conceptualization, Funding acquisition, Project administration, Supervision, Writing – original draft.
